# Impact of smoking on stroke outcome after endovascular treatment

**DOI:** 10.1371/journal.pone.0194652

**Published:** 2018-05-02

**Authors:** Rascha von Martial, Jan Gralla, Pasquale Mordasini, Marwan El Koussy, Sebastian Bellwald, Bastian Volbers, Rebekka Kurmann, Simon Jung, Urs Fischer, Marcel Arnold, Hakan Sarikaya

**Affiliations:** 1 Department of Neurology, Inselspital, University Hospital Bern and University of Bern, Bern, Switzerland; 2 Department of Diagnostic and Interventional Neuroradiology, Inselspital, University Hospital Bern and University of Bern, Bern, Switzerland; Osaka University Graduate School of Medicine, JAPAN

## Abstract

**Background:**

Recent studies suggest a paradoxical association between smoking status and clinical outcome after intravenous thrombolysis (IVT). Little is known about relationship between smoking and stroke outcome after endovascular treatment (EVT).

**Methods:**

We analyzed data of all stroke patients treated with EVT at the tertiary stroke centre of Berne between January 2005 and December 2015. Using uni- and multivariate modeling, we assessed whether smoking was independently associated with excellent clinical outcome (modified Rankin Scale (mRS) 0–1) and mortality at 3 months. In addition, we also measured the occurrence of symptomatic intracranial hemorrhage (sICH) and recanalization.

**Results:**

Of 935 patients, 204 (21.8%) were smokers. They were younger (60.5 vs. 70.1 years of age, p<0.001), more often male (60.8% vs. 52.5%, p = 0.036), had less often from hypertension (56.4% vs. 69.6%, p<0.001) and were less often treated with antithrombotics (35.3% vs. 47.7%, p = 0.004) as compared to nonsmokers. In univariate analyses, smokers had higher rates of excellent clinical outcome (39.1% vs. 23.1%, p<0.001) and arterial recanalization (85.6% vs. 79.4%, p = 0.048), whereas mortality was lower (15.6% vs. 25%, p = 0.006) and frequency of sICH similar (4.4% vs. 4.1%, p = 0.86). After correcting for confounders, smoking still independently predicted excellent clinical outcome (OR 1.758, 95% CI 1.206–2.562; p<0.001).

**Conclusion:**

Smoking in stroke patients may be a predictor of excellent clinical outcome after EVT. However, these data must not be misinterpreted as beneficial effect of smoking due to the observational study design. In view of deleterious effects of cigarette smoking on cardiovascular health, cessation of smoking should still be strongly recommended for stroke prevention.

## Introduction

Smoking is an independent risk factor for ischemic stroke especially in younger people.[1] The World Health Organization (WHO) estimates that smoking causes about six million deaths worldwide per year, with increasing incidences especially in developing countries.[2] Though detrimental effects on vascular health, recent studies report better clinical outcome in smokers treated with thrombolysis, which is termed as “smoking paradox”. It was first described in patients with myocardial infarction undergoing intravenous thrombolysis (IVT).[3, 4] Similar associations have also been reported for smoking, favorable 3-month outcome and recanalization in stroke patients treated with IVT.[5] Little is known about relationship between smoking status and stroke outcome after endovascular treatment (EVT), which is increasingly preferred for treatment of severe strokes with large artery occlusion.[6–9]

## Methods

This study was based on the Bernese stroke center database, a systematic prospective registry of consecutive patients with ischemic stroke treated at the Stroke Center of University Hospital of Berne, Switzerland. It was approved by the Local Ethics Committee Bern. For this study, we analyzed all stroke patients who underwent EVT (mechanical thrombectomy and/or intraarterial thrombolysis (IAT) with urokinase) between January 2005 and December 2015. The following variables and stroke risk factors were prospectively collected as defined previously:[10–12] age, sex, smoking status, antithrombotic medication at stroke onset, diabetes mellitus, hypertension, hypercholesterolemia, functional independence before stroke (according to modified Rankin Scale (mRS)), stroke etiology according to the Trial of ORG 10172 in Acute Stroke Treatment (TOAST) criteria, stroke onset-to-treatment time as well as blood pressure and blood glucose level obtained on admission. Patients were classified as smokers when they reported active cigarette use. Severity of the neurological deficit was assessed by a stroke neurologist at admission by using the National Institutes of Health Stroke Scale (NIHSS) score.[13] All patients underwent immediate brain imaging by magnetic resonance imaging (MRI) or computer tomography (CT). We performed EVT according to international and institutional guidelines as described previously. Patients were treated with intra-arterial urokinase, mechanical interventions, or both. Patients within time window of 3 to 4.5 hours were additionally treated with IVT.[14–16] Vessel occlusion and collateral supply was monitored with conventional angiography by an interventional neuroradiologist.[17, 18] Localization of arterial occlusion was categorized depending on size as large vessel (internal carotid artery including carotid-T) vs. medium vessel occlusion (e.g. M1-segment of middle cerebral artery and basilar artery). Collaterals were classified as poor, if none or minimal leptomeningeal anastomoses were visualized and no or minimal filling of the occluded vessel territory was seen, and good, if leptomeningeal anastomoses filled the occluded vessel territory by more than half.[17, 18] All patients treated with EVT were admitted to intermediate or intensive care unit or stroke unit for at least 24 hours. All patients underwent brain imaging with MRI or CT 24 hours after intervention and in any case of clinical deterioration. Symptomatic intracranial hemorrhage (sICH) was defined according to ECASS II criteria.[19] Status of recanalization was assessed immediately after EVT by using the Thrombolysis in Myocardial infarction (TIMI) score on angiography.[20, 21] Partial or complete recanalization (TIMI scores 2 and 3) were classified as successful recanalization.[22–24] Primary endpoints were excellent clinical outcome at 3 months after stroke (mRS 0 or 1), death within 3 months (mRS 6) and the occurrence of sICH. Secondary endpoint was the frequency of successful recanalization after EVT. The endpoints were prospectively assessed during hospital stay and/or 3-month outpatient visits.

### Statistical analyses

We compared demographic and baseline characteristics between smokers and nonsmokers by using Pearson’s chi-squared test for categorial variables and two-tailed t-test for continuous variables (or Mann-Whitney U test for skewed distribution). The independent effect of smoking status on endpoints was assessed in multivariate analyses using blockwise regression model. Multivariable adjustment was applied for significant baseline differences between smokers and nonsmokers. Level of significance was set to p < 0.05. Furthermore, we performed subgroup analyses for patients treated with mechanical thrombectomy only and for those undergoing intra-arterial thrombolysis (IAT) with urokinase (± mechanical thrombectomy), respectively. The level of statistical significance was set to 0.05. Statistical analysis was made using the SPSS Version 21.0.

## Results

### Baseline characteristics

A total of 935 patients were eligible for this study. Of these, 204 (21.8%) were current smokers. Thrombolysis with IAT was performed in 405 (43.3%) patients, while 285 (30.5%) underwent mechanical thrombectomy only and 245 (26.2%) were treated with intra-arterial urokinase in combination with intravenous alteplase. The main baseline characteristics of the 2 groups are detailed in Table 1. Compared with nonsmokers, smokers were younger and more often male. They suffered less often from arterial hypertension, more often from hypercholesterolemia and were less often treated with antithrombotics. On admission, NIHSS and systolic blood pressure were significantly lower in smokers. Large-artery atherosclerosis was more often the underlying stroke reason in smokers, whereas cardioembolism was the leading stroke cause in nonsmokers. Baseline imaging revealed more often an occlusion of large arteries in smokers than in the counterpart. In analogy, pure mechanical thrombectomy was more often performed in smokers, whereas nonsmokers underwent more often IAT.

**Table 1:** Baseline characteristics in smoking and nonsmoking stroke patients.

	All Patients (n = 935)	Smokers (n = 204)	Nonsmokers (n = 731)	p
Mean age ± SD, years	68 ± 13.9	60.5 ± 12.1	70.1 ± 13.7	<0.001
Male gender	508/935 (54,3)	124/204 (60.8)	384/731 (52.5)	0.036
Diabetes mellitus	152/935 (16.3)	26/204 (12.7)	126/731 (17.2)	0.124
Arterial hypertension	624/935 (66.7)	115/204 (56.4)	509/731 (69.6)	<0.001
Hypercholesterolemia	527/925 (57)	128/203 (63.1)	399/722 (55.3)	0.048
Antithrombotic pretreatment	342/761 (44.9)	60/170 (35.3)	282/591 (47.7)	0.004
Functional independency before stroke (mRS 0–2)	890/934 (95.3)	198/204 (97.1)	692/730 (94.8)	0.177
NIHSS at admission, median (IQR)	15.0 (10–19)	14.0 (9–18)	15.0 (10–20)	0.024
Mean systolic blood pressure/mmHg ± SD	154 ± 27.5	149 ± 26.8	156 ± 27.5	0.006
Mean glucose/mmol/l ± SD	7.18 ± 2.29	6.87 ± 2.16	7.26 ± 2.36	0.062
Median time to treatment/min (IQR)	274 (213–355)	280 (224–359)	273 (210–351)	0.436
Stroke etiology (TOAST)				<0.001
- large artery atherosclerosis	139/932 (14.9)	49/203 (24.1)	90/729 (12.3)	
- cardioembolic	409/932 (43.9)	60/203 (29.6)	349/729 (47.9)	
- others, n (%)	45/932 (4.8)	10/203 (4.9)	35/729 (4.8)	
- undetermined, n (%)	339/932 (36.4)	84/203 (41.4)	255/729 (35)	
Localization of arterial occlusion				0.01
- large vessel, n (%)	200/817 (24.5)	54/168 (32.1)	146/649 (22.5)	
- medium vessel, n (%)	617/817 (75.5)	114/168 (67.9)	503/649 (77.5)	
Good collaterals, n (%)	302/792 (38.1)	62/158 (39.2)	240/634 (37.9)	0.828
Type of treatment				0.031
- IAT	405/935 (43.3)	73/204 (35.8)	332/731 (45.4)	
- IAT + IVT	245/935 (26.2)	56/204 (27.5)	189/731 (25.9)	
- mechanical thrombectomy	285/935 (30.5)	75/204 (36.8)	210/731 (28.7)	

Results are expressed in n/N (%) unless otherwise denoted

IA denotes intra-arterial thrombolysis with urokinase, IQR interquartile range, IV intravenous thrombolysis with alteplase, min minute, mRS modified Rankin Scale, NIHSS National Institutes of Health Stroke Scale, SD standard deviation, TOAST Trial of Org 10172 in Acute Stroke Treatment.

## Outcomes

**Fig 1:**
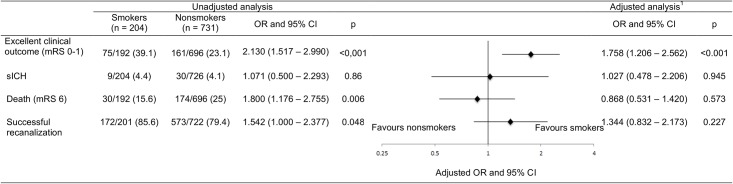
Outcomes in smoking vs. non-smoking stroke patients after endovascular treatment. Results are expressed in n/N (%). CI confidence interval, mRS modified Rankin Scale, OR odds ratio, sICH symptomatic intracranial hemorrhage. ^1^ adjusted for age, sex, NIHSS on admission, systolic blood pressure on admission, stroke etiology, localization of occluded vessel, hypercholesterolemia and use of antithrombotic pretreatment.

Outcomes are summarized in Fig 1. At 3 months, smokers showed significantly higher rates of excellent clinical outcome and lower mortality rates as compared to nonsmokers (39.1% vs. 23.1%, p < 0.001 and 15.6% vs. 25%, p = 0.006, respectively), whereas the risk of sICH was comparable in both groups (4.4% vs. 4.1%, p = 0.006). The frequency of successful recanalization was higher in smokers than in nonsmokers (85.6% vs. 79.4%, p = 0.048). In line with this, stroke severity measured by NIHSS at 24 hours after treatment was significantly lower in smokers as compared to the counterpart (7.0 vs. 10.0, p = 0.005).

Details of multivariate analyses are shown in S1 Table. Adjustment was made for following variables with statistically significant differences at baseline: age, sex, NIHSS and systolic blood pressure at admission, smoking status and antithrombotics before stroke onset, stroke etiology according to TOAST criteria, localization of occluded vessel and hypercholesterolemia. Multivariate analyses revealed current smoking status as an independent predictor of excellent clinical outcome (Odds ratio (OR) 1.758, 95% confidence interval (95% CI) 1.206–2.562, p < 0.001) together with age and baseline NIHSS score. Mortality at 3 months was also independently associated with age and baseline NIHSS, but not with smoking status (OR 0.868, 95% CI 0.531–1.420, p = 0.573). None of the variables was independently associated with sICH, this was also true for smoking status (OR 1.027, 95% CI 0.478–2.206, p = 0.945). Hypercholesterolemia and large vessel occlusion were identified as independent predictors of recanalization after EVT, whereas no association was observed with smoking status (OR 1.344, 95% CI 0.832–2.173, p = 0.227).

We additionally performed subgroup analyses with focus on patients undergoing mechanical thrombectomy only (n = 285) and those treated with intra-arterial urokinase (n = 405). Logistic regression analyses included age, NIHSS at admission, large vessel occlusion and smoking status as covariates. The results are shown in Tables 2 and 3. Smokers treated with pure mechanical thrombectomy had significantly higher rates of excellent clinical outcome at 3 months as compared to nonsmokers (37.1% vs. 19%, p = 0.002), while no differences were detected regarding mortality at 3 months, sICH and recanalization. Logistic regression analysis revealed an independent association for age and NIHSS (OR 0.199, 95% CI 0.942–0.990, p = 0.006 and OR 0.856, 95% CI 0.803–0.914, p < 0.001, respectively), but not for smoking (OR 1.604, 95% CI 0.724–3.551, p = 0.244). Smokers treated with intra-arterial urokinase reached also higher rates of excellent clinical outcome (39.4% vs. 20.9%, p = 0.001) and tended to lower mortality risk (15.5% vs. 24.2%, p = 0.058). The following variables were strongly associated with excellent clinical outcome: age (OR 0.973, 95% CI 0.954–0.991, p = 0.004), NIHSS (OR 0.910, 95% CI 0.872–0.950, p < 0.001), smoking (OR 2.412, 95% CI 1.245–4.671, p = 0.009).

**Table 2:** Outcomes after mechanical thrombectomy only in smoking vs. nonsmoking patients.

	All patients (n = 285)	Smokers (n = 75)	Nonsmokers (n = 210)	p
Excellent clinical outcome (mRS 0–1)	63/265 (23.8)	26/70 (37.1)	37/195 (19)	0.002
Death	68/265 (25.7)	14/70 (20)	54/195 (27.7)	0.206
sICH	13/284 (4.6)	6/75 (8)	7/209 (3.3)	0.098
Successful recanalization	235/277 (84.8)	64/74 (86.5)	171/203 (84.2)	0.644

Results are expressed in n/N (%)

mRS denotes modified Rankin Scale, NIHSS National Institutes of Health Stroke Scale and sICH, symptomatic intracranial hemorrhage

**Table 3:** Outcomes after IAT with urokinase in smoking vs. nonsmoking patients.

	All patients (n = 405)	Smokers (n = 73)	Nonsmokers (n = 332)	p
Excellent clinical outcome (mRS 0–1)	96/396 (24.2)	28/71 (39.4)	68/325 (20.9)	0.001
Death	96/396 (24.2)	11/71 (15.5)	85/325 (26.2)	0.058
sICH	20/402 (5)	2/73 (2.7)	18/329 (5.5)	0.332
Successful recanalization	288/402 (71.6)	53/71 (74.6)	235/331 (71)	0.536

Results are expressed in n/N (%)

mRS denotes modified Rankin Scale, NIHSS National Institutes of Health Stroke Scale and sICH, symptomatic intracranial hemorrhage

## Discussion

This study suggests that current smoking may be an independent predictor of excellent clinical outcome in ischemic stroke treated with EVT. To date, many studies investigated the relationship between smoking status and outcome after IVT in stroke patients and reported conflicting results.[5, 25–28] However, data on EVT are scarce. Meseguer and colleagues showed that intra-arterial administration of rtPA independently predicts arterial recanalization in smokers, but had no significant impact on clinical endpoints such as favorable outcome, mortality or sICH.[29] However, the cohort size was rather small as compared to our study (227 vs. 935 patients). We are not aware of further studies assessing the relationship between smoking and stroke outcome after EVT.

Considering differences in baseline characteristics is essential for discussion of “smoking paradox” in stroke patients. In line with literature, smokers in our study were significantly younger, more likely to be male and suffered less often from atrial fibrillation and arterial hypertension than nonsmokers.[5, 26, 27, 29] These imbalances could partially explain the better clinical outcomes in favor of smokers, as older age and male gender have been shown to be associated with worse clinical outcomes.[30–33] In line with this, Hussein et al. suggested that smoking paradox may be mainly related to differences in age.[25] In addition, Tong et al. recently reported that noncardioembolic stroke may be independently related to good outcome in smoking patients treated with IVT.[34] Cardioembolism was the underlying stroke reason for almost every second non-smoking patient in our study. Cardioembolic stroke due to arterial fibrillation is considered to be an independent risk factor for large territorial infarcts, hemorrhagic transformation and unfavorable outcome after stroke.[35–37] Furthermore, stroke severity at admission (measured by NIHSS) tended to be lower in smokers which may also have contributed to differences in outcome.[38, 39]

Mechanical thrombectomy was more often used in smokers (36.8% vs. 28.7%), whereas IAT was the preferred treatment in non-smokers (45.4% vs. 35.8%). We assume, that these differences in treatment may primarily be related to higher proportion of large vessel occlusions in smokers.

The relationship between favorable outcome and smoking status after thrombolysis has also been reported in stroke patients treated with IVT.[5] Other studies could not detect an association between outcome and smoking habits though higher rates of recanalization in smokers.[26, 29] The conflicting results may be related to differences in study size as the number of included patients reported in studies range from 94 to 5383.[5, 25] Furthermore, location of vessel occlusion may have played an important role. Smoking patients suffered more frequently from large artery occlusion, which was independently associated with recanalization in this study. The association between higher recanalization rates and proximal vessel occlusion in EVT has already been reported in a large cohort with anterior circulation stroke.[40] In contrary, large vessel occlusion is inversely related to recanalization and outcome in stroke patients treated with IVT.[41–43] Hypercholesterolemia was another independent predictor of recanalization in our study. Vanacker et al. reported an independent association between hypercholesterolemia and spontaneous recanalization in patients with ischemic stroke.[44] Galimanis et al. also observed higher recanalization rates in stroke patients with hypercholesterolemia after EVT.[40] Antioxidant effects of lipids or lower rates of concomitant infections were discussed as potential explanations.[45, 46] Pleiotropic effects of statins in these patients may also be of importance.[47]

An increased efficacy of rtPA in smokers has been reported before with respect to different rates of recanalization depending on thrombus composition.[28, 29] Smoking patients are assumed to have an increased hematocrit, platelet activation and aggregation, vasoconstriction and circulating fibrinogen.[48–50] Thus, smokers may have more thrombogenic than atherogenic vessel occlusion, whereas nonsmokers may have more likely vessel occlusion due to ruptured or ulcerated plaque with platelet-rich clot.[51] However, we could not prove a strong association between smoking status and recanalization after EVT. Ali et al. reported a lower in-hospital mortality in smoking stroke patients irrespective of thrombolysis and discussed a greater susceptibility of thrombi to therapeutic as well as to spontaneous thrombolysis due to the different thrombus compositions.[51] Our subgroup analyses suggest that smoking may have a greater impact on clinical outcome in patients treated with IA urokinase than in those treated with pure mechanical thrombectomy. This finding could be in line with the abovementioned hypothesis of increased susceptibility of thrombi to fibrinolytic drugs in smokers. Mortality among smokers was also lower in our study, but the association lost significance after adjusting for confounders. Smokers may be supposed to have a better cerebral collateral supply as a further explanation for paradoxical association with clinical outcome, but collateral supply did not differ between groups in our study. Still, smokers may be better preconditioned on ischemia due to increased plasma levels of carbon monoxide and episodic hypoxia.[52] Smoking status had no impact on risk of sICH after treatment in this study, which is in line with literature.[5, 27, 29]

The main strength of this study is the large cohort size, which allows adjustment for potential confounders and decreases the odds for false-positive results. This is to our best knowledge the largest study assessing relationship between EVT and smoking status. Furthermore, data quality was high as both clinical and radiological data were systematically and prospectively collected at baseline and during 3-month follow-up by certified neurologists and neuroradiologists. In addition, we aimed at comprehensive data collection including location of vessel occlusion, recanalization and collateral supply, which were not considered in former studies.

We are aware of several limitations. First, this was an observational study with an inherent risk of treatment bias which may not be completely removed though multivariate model. As consequence, a firm conclusion about causality between smoking and outcome is prohibited. Second, we did not record quantity of smoking exposure, leading to a heterogeneity in our smoking cohort and hindering a differentiation of heavy vs. mild smokers. A dose-dependent relation between smoking and blood viscosity with increase of plasma-fibrinogen level has been reported.[53] Third, there may be some hidden confounders which were not included in our study such as mode and intensity of rehabilitation, socio-economic status and medical complications. Furthermore we did not adjust for other prognostic factors such as time to recanalization or radiological scores of stroke severity. Fourth, current guidelines recommend the use of TICI (Thrombolysis In Cerebral Infarction) study for assessment of arterial recanalization.[54] We used TIMI scale for historical reasons, because recommendation for TICI was published towards the end of our study period. Fifth data were collected over ten years during which a great evolution on stroke treatment was taking place. Especially recanalization techniques and acute stroke care like stroke unit care and early rehabilitation might influence outcome in stroke patients. Nevertheless endovascular stroke therapy has a long tradition in our center and has been systematically performed. The publication of the major randomised controlled trials on EVT did not change our treatment approach. Furthermore the main question was to determine differences between smokers and nonsmokers. Those two groups were treated with same procedures and were affected in a similar way of mentioned changes. Finally, we did not measure changes in smoking habits (e.g. cessation of smoking) after stroke which may also have influenced the 3-month outcome.

## Conclusion

In summary, our data indicate that smoking in stroke patients may be independently associated with excellent clinical outcome after EVT. Though strong relationship in multivariable analyses, significant differences in baseline characteristics still needed to be considered together with different pathophysiological mechanisms. A conclusion on causality is not reliable due to the observational study design. Thus, these data must not be misinterpreted as a beneficial effect of smoking. In view of deleterious effects of cigarette smoking on cardiovascular health, cessation of smoking should still be strongly recommended for both primary and secondary stroke prevention

## Supporting information

S1 Table Blockwise-backwards regression analyses for favorable outcome, death after 3 months sICH and recanalization. CI confidence interval, IQR denotes interquartile range, mRS modified Rankin Scale, NIHSS National Institutes of Health Stroke Scale, OR odds ratio, sICH, symptomatic intracranial hemorrhage.
(DOCX)
