# Supplementary material for: Impact of smoking on stroke outcome after endovascular treatment
Source: PLoS One. 2018 May 2;13(5):e0194652. doi: 10.1371/journal.pone.0194652 (PMC5931491; doi:10.1371/journal.pone.0194652)
Supplement: S1 Table — CI confidence interval, IQR denotes interquartile range, mRS modified Rankin Scale, NIHSS National Institutes of Health Stroke Scale, OR odds ratio, sICH, symptomatic intracranial hemorrhage. (DOCX) [file pone.0194652.s001.docx]

**S1 Table.** **Blockwise-backwards regression analyses for favorable outcome, death after 3 months sICH**

**and recanalization**

|  | | | | | | | | | OR and 95% CI | p |
| --- | --- | --- | --- | --- | --- | --- | --- | --- | --- | --- |
| **Excellent clinical outcome**  *First step (Nagelkerke R Square 0.198)* | | | | | | | | | 0.975 (0.961 – 0.990)  0.906 (0.622 – 1.319)  0.901 (0.873 – 0.929)  0.998 (0.991 – 1,006)  0.621 (0.355 – 1.086)  1.397 (0.940 – 2.077)  0.657 (0.437 – 0.989)  1.639 (1.062 – 2.527) | 0.001  0.606  <0.001  0.640  0.95  0.98  0.044  0.025 |
|  | | | age  male gender  NIHSS at admission  systolic blood pressure at admission  stroke etiology  hypercholesterolemia  antithrombotic use  smoking | | | | | |  |  |
| *Last step (Nagelkerke R Square 0.186)* | | | | | | | | | |  |
|  | | | | | age  NIHSS on admission  smoking | | | | 0.978 (0.967 – 0.989)  0.895 (0.872 – 0.920)  1.758 (1.206 – 2.562) | <0.001  <0.001  <0.001 |
| **Death (mRS 6)**  *First step (Nagelkerke R Square 0.250)* | | | | | | | | | 1.064 (1.040 – 1.088)  1.135 (0.745 – 1.728)  1.098 (1.068 – 1.128)  1.000 (0.993 – 1.008)  1.186 (0.597 – 2.355)  0.802 (0.521 – 1.235)  1.352 (0.872 – 2.096)  0.925 (0.512 – 1.670) | <0.001  0.556  <0.001  0.925  0.626  0.317  0.177  0.796 |
|  | | | | | | | | age  male gender  NIHSS at admission  systolic blood pressure at admission  stroke etiology  hypercholesterolemia  antithrombotic use  smoking |  |  |
| *Last step (Nagelkerke R Square 0.199)* | | | | | | | | | 1.053 (1.036 – 1.070)  1.093 (1.069 – 1.117)  0.868 (0.531 – 1.420) | <0.001  <0.001  0.573 |
|  | | | | | | | age  NIHSS on admission  smoking | |  |  |
| **sICH**  *First step (Nagelkerke R Square 0.047)* | | | | | | | | | 0.985 (0.953 – 1.019)  0.647 (0.280 – 1.499)  1.009 (0.958 – 1.063)  0.995 (0.979 – 1.011)  1.563 (0.531 – 4.600)  0.960 (0.416 – 2.216)  1.965 (0.826 – 4.673)  1.029 (0.401 – 2.644) | 0.386  0.310  0.729  0.521  0.417  0.924  0.126  0.952 |
|  | | | | | | age  male gender  NIHSS at admission  systolic blood pressure at admission  stroke etiology  hypercholesterolemia  antithrombotic use  smoking | | |  |  |
| *Last step (Nagelkerke R Square 0.009)* | | | | | | | | | 0.585 (0.296 – 1.154)  1.027 (0.478 – 2.206) | 0.122  0.945 |
|  | | | | male gender  smoking | | | | |  |  |
| **Recanalization**  *First step (Nagelkerke R Square 0.032)* | | | | | | | | | |  |
|  | | age  male gender  NIHSS at admission  systolic blood pressure at admission  stroke etiology  hypercholesterolemia  antithrombotic use  localization of arterial occlusion  smoking | | | | | | | 0.988 (0.968 – 1.009)  0.832 (0.516 – 1.343)  1.019 (0.986 – 1.054)  0.998 (0.989 – 1.008)  0.702 (0.318 – 1.548)  1.469 (0.896 – 2.406)  0.756 (0.456 – 1.256)  0.634 (0.345 – 1.165)  1.189 (0.624 – 2.266) | 0.266  0.451  0.255  0.714  0.380  0.127  0.281  0.142  0.599 |
| *Last step (Nagelkerke R Square 0.035)* | | | | | | | | | | 0.012  0.006  0.227 |
|  | hypercholesterolemia  localization of arterial occlusion  smoking | | | | | | | | 1.583 (1.105 –2.267)  0.508 (0.314 – 0.820)  1.344 (0.832 – 2.173) |  |

CI denotes confidence interval, IQR interquartile range, mRS modified Rankin Scale, NIHSS National Institutes of Health Stroke Scale, OR odds ratio, sICH, symptomatic intracranial hemorrhage
